# Thinking outside the blood: Perspectives on tissue-resident *Trypanosoma brucei*

**DOI:** 10.1371/journal.ppat.1009866

**Published:** 2021-09-16

**Authors:** Nathan P. Crilly, Monica R. Mugnier

**Affiliations:** 1 Department of Molecular Microbiology and Immunology, Johns Hopkins Bloomberg School of Public Health, Baltimore, Maryland, United States of America; 2 Department of Molecular and Comparative Pathology, Johns Hopkins School of Medicine, Baltimore Maryland, United States of America; Joan and Sanford I Weill Medical College of Cornell University, UNITED STATES

## Abstract

*Trypanosoma brucei* is a protozoan parasite that causes human and animal African trypanosomiases (HAT and AAT). In the mammalian host, the parasite lives entirely extracellularly, in both the blood and interstitial spaces in tissues. Although most *T*. *brucei* research has focused on the biology of blood- and central nervous system (CNS)-resident parasites, a number of recent studies have highlighted parasite reservoirs in the dermis and adipose tissue, leading to a renewed interest in tissue-resident parasite populations. In light of this renewed interest, work describing tissue-resident parasites can serve as a valuable resource to inform future investigations of tissue-resident *T*. *brucei*. Here, we review this body of literature, which describes infections in humans, natural hosts, and experimental animal models, providing a wealth of information on the distribution and biology of extravascular parasites, the corresponding immune response in each tissue, and resulting host pathology. We discuss the implications of these studies and future questions in the study of extravascular *T*. *brucei*.

## Introduction

*Trypanosoma brucei* is a unicellular kinetoplastid pathogen and causative agent of human African trypanosomiasis (HAT) and animal African trypanosomiasis (AAT), 2 emerging and neglected parasitic diseases of medical and veterinary importance. *T*. *brucei* is divided into 3 major subspecies: *T*. *b*. *gambiense* (Tbg), *T*. *b*. *rhodesiense* (Tbr), and *T*. *b*. *brucei* (Tbb). All 3 subspecies infect animals, while Tbg and Tbr cause human disease [1,2]. *T*. *brucei* is restricted to sub-Saharan Africa and is transmitted by the bite of a tsetse fly vector [2,3].

Clinically, HAT is highly variable but is generally characterized by 2 stages. Stage 1, the hemolymphatic stage, consists of waxing and waning flulike symptoms, believed to correspond to waves of parasitemia [4]. Stage 2, the neurological stage, is characterized by a spectrum of neurological symptoms due to meningoencephalitis and cerebral edema [4]. AAT, which affects a wide variety of domestic and wild mammals, shares some clinical features with HAT but is characterized primarily by weight loss, with or without acute febrile signs or chronic neurological illness [5].

**Fig 1:**
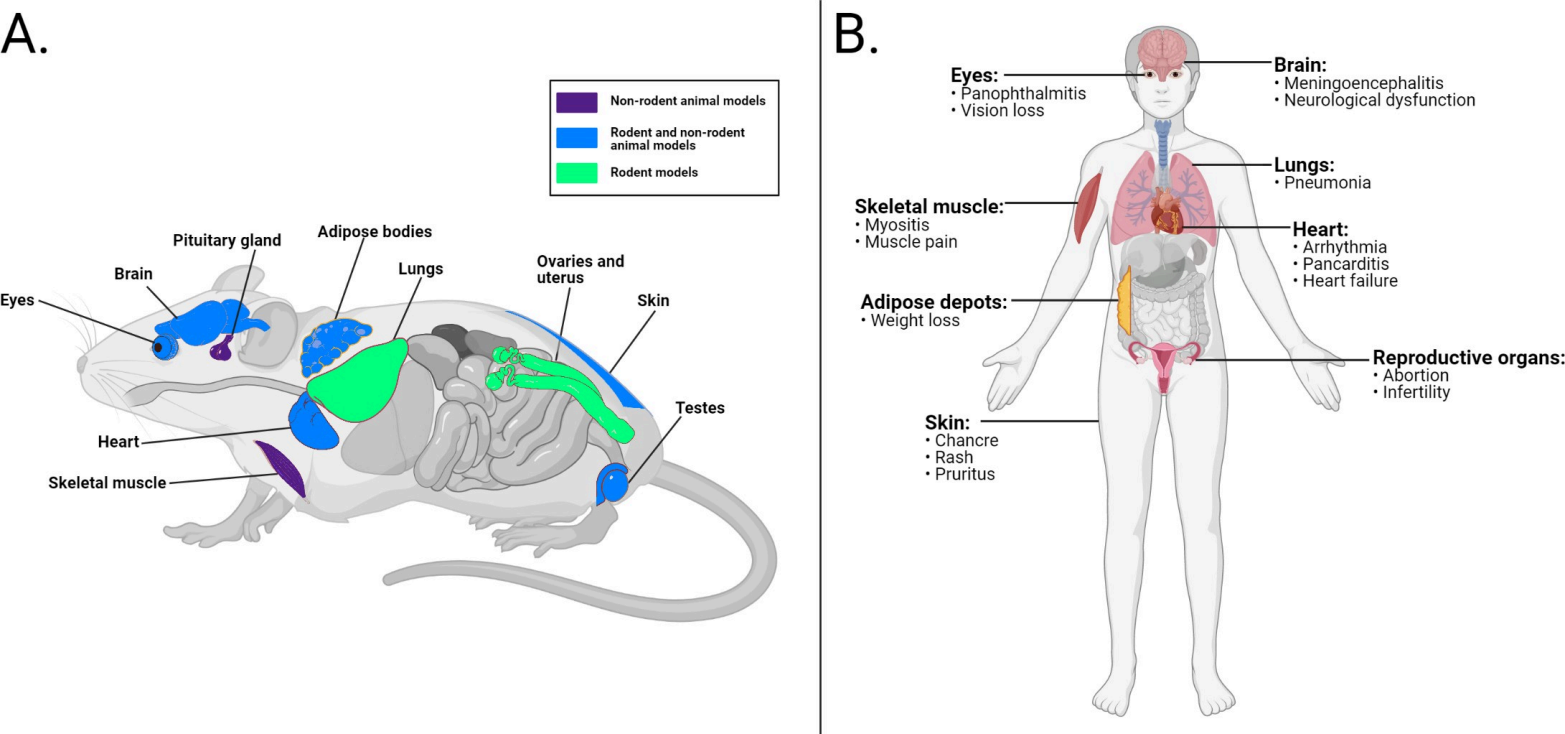
Extravascular trypanosomes and organ-specific symptoms in humans and animals. **(A)** Organs in which extravascular trypanosomes have been observed in animal models. (**B)** Organ-specific symptoms of HAT, which may be associated with extravascular trypanosomes. (Created with Biorender.com). HAT, human African trypanosomiasis.

Since at least as early as 1910, there have been an abundance of descriptive and observational studies that have identified extravascular trypanosomes in multiple organs of humans and animals (Fig 1A) [6–14]. These tissue-resident parasites may be implicated in human and veterinary disease and are thought to contribute to several clinical aspects of African trypanosomiasis, such as heart disease, weight loss, and vision loss (Fig 1B) [11,13,15,16]. Despite the large number of observational studies on extravascular trypanosomes, there has been limited investigation into extravascular parasites at the molecular or mechanistic level. Recent findings, however, have highlighted significant parasite reservoirs in the adipose tissue, testes, and skin [17–20], leading to a renewed interest in extravascular parasites as a subject of research.

In light of the recent increase in interest in extravascular parasites, the rich history of literature on tissue-resident parasites and their accompanying pathology is a valuable but largely untapped resource. This body of literature provides a wealth of information on the distribution and biology of extravascular parasites, the corresponding immune response in each tissue, and resulting host pathology. These observations, gathered from infections in humans, natural hosts, and experimental animal models, can serve to inform future studies of tissue-resident *T*. *brucei*. Here, we provide a review of the literature on tissue-resident parasites and associated pathology outside of the central nervous system (CNS). Although CNS injury plays a major role in HAT, the pathogenesis and pathology of *T*. *brucei* within the CNS have been reviewed elsewhere [3,21].

### Skin and subcutis

Tissue-resident parasites in the skin and subcutis have been relatively well studied and may play an important role in pathogenesis and transmission. A variety of dermatological symptoms have been reported in association with HAT, including rashes, pruritus, and dermatitis [22]. At least one dermatological symptom is reported in up to 94% of patients during early and/or late disease [22,23]. One of the best described dermatological lesions of HAT and AAT is the trypanosomal chancre, which highlights the importance of the skin during early infection. The trypanosomal chancre is a nodule of proliferating parasites and associated inflammation that can form in the skin at the site of the tsetse fly bite shortly after infection [24–26]. The presence or absence of the chancre is highly variable and depends on both the infecting subspecies of *T*. *brucei* and the origin of the patient. The chancre is observed more frequently in travelers than in residents of endemic areas and is more common in cases of Tbr than Tbg, being recorded in fewer than 5% of native residents with Tbg and up to 90% of travelers with Tbr [22,24,27,28]. Histologically, the chancre is characterized by large numbers of extravascular parasites and associated inflammation [24]. Parasites within the chancre are reported to have an affinity for collagen bundles, a feature that has also been observed in multiple other tissues and species [7,12,29–31]. The host response within the chancre is comprised mainly of perivascular lymphoplasmacytic and histiocytic infiltrates, with proliferation of fibroblasts and endothelial cells. When present, the chancre appears and then recedes within weeks following infection [24,32].

Until recently, early postinfection events in the skin had been best characterized by a pair of experiments in goats [12,25]. Following intradermal infection of goats by Tbb, dermal parasites rapidly proliferated in the chancre at the site of infection and underwent transformation from metacyclic to bloodstream forms [12,25]. As in humans, parasites in the chancre exhibited a possible affinity for collagen: 20% of parasites within chancres were localized to collagen bundles, while the remainder occupied interstitial spaces or were closely associated with fibroblasts [12]. Findings in the goat also suggested that the chancre may be a unique environment that affects parasite morphology and viability. A minority of dermal parasites exhibited unique morphology, including small mitochondria, poorly differentiated rough endoplasmic reticulum, and uniformly sized glycosomes. These changes hint at metabolic changes in parasites in the skin, potentially reflecting an adaptation to the local nutrient environment [12]. Approximately 50% to 75% of dermal parasites were lysed, although dead parasites were not associated with any specific adjacent inflammatory cells [12]. These observations suggest that, while the skin is important for establishing early infection, it can still be a hostile environment for trypanosomes, and dermal parasites may exhibit adaptations to survive in the dermis. However, it is uncertain if these findings apply to other mammalian hosts.

More recently, early postinfection events have been studied in mice intradermally infected with Tbb [30]. Although findings were comparable to those in goats, there were some differences in parasite location and viability between the 2 species. Following intradermal infection of mice, parasites could be detected in skin prior to detection in lymph and blood [30]. Similar to in goats, skin-resident trypanosomes in mice rapidly proliferated in the dermis [30]. However, there were differences in parasite localization between mice and goats. In mice, skin-resident trypanosomes interacted with dermal collagen bundles, as seen in both goats and humans. Unlike in goats, however, dermal trypanosomes in mice also interacted with adipocytes and peri-adipocyte connective tissue. There was also a substantial difference in parasite survival between mice and goats. In goats, half of dermal parasites were lysed at 3 days postinfection, whereas in mice, fewer than 1% of dermal parasites were nonviable after 3 days [30]. These differences suggest that although skin tropism is conserved between mammalian hosts, the behavior of skin-resident parasites and the local immune response in the skin may vary between host species.

In addition to early events, there is recent evidence that the skin is an important anatomical reservoir for parasites during chronic infection and that skin tropism may play a role in transmission to the tsetse fly vector. Recently, 2 studies have identified Tbg parasites in the skin of unconfirmed seropositive individuals, demonstrating the potential importance of the skin as a reservoir during long-term infection [18,23]. In support of these findings, a recent study found that Tbb parasites rapidly established a quiescent population when cultured in artificial human skin, suggesting that skin-resident parasites could establish a long-term reservoir in the skin [33]. In addition, in one recent mouse study, skin-resident Tbb parasites were sufficient to initiate a tsetse fly infection in the absence of parasitemia [18]. Together, these findings suggest that skin tropism may play a major and unappreciated role in maintaining long-term infection and improving parasite transmissibility. The potential importance of the skin reservoir has been highlighted by recent mathematical models, which have predicted that skin-resident parasites could stymie HAT elimination efforts due to continuing transmission from the skin of asymptomatic individuals, who are difficult to detect by conventional methods [34,35].

### Lymphoid organs

Following infection, parasites can use lymphatics and draining lymph nodes to spread to the bloodstream [30,36]. In humans, lymph node enlargement is common, occurring in up to 95% of patients during acute disease [22,27]. Parasites can often be found in the lymph fluid by fine needle aspiration, though it is unclear whether they inhabit lymphoid tissue [37]. Unfortunately, there is a lack of human lymph node biopsies from active HAT cases, and extravascular parasites have not been identified in the few lymph nodes, which have been examined [21,38,39]. Thus, the presence or absence of lymphoid organ-resident parasites is uncertain [31]. In general, human lymph nodes exhibit increased macrophages and plasma cells, while other lymphocytes are reduced in number, indicating a chronic immune response resulting in immune exhaustion [21].

As in humans, parasites have not been definitively observed within the extravascular spaces of lymphoid organs in animal models. In rabbits experimentally infected with Tbb, parasites have been observed in the red pulp of the spleen, but it is uncertain whether they were extravascular or within vascular channels [40]. In sheep experimentally infected with Tbb and monkeys experimentally infected with Tbg, parasites have been observed in connective tissue surrounding the spleen and lymph nodes [6,7], but they have not been observed within the interstitial spaces of the organs themselves.

The host response within lymphoid organs is consistent across most animal models. In sheep, dogs, rabbits, and deer mice experimentally infected with Tbb, the spleen and lymph nodes have been enlarged, with edema, prominent germinal centers, plasmacytic and histiocytic infiltrates, and lymphocyte depletion [7,8,40,41]. As in humans, these changes are characteristic of a chronic immune response that progresses to immune exhaustion.

Overall, most data suggest that parasites observed in the lymph nodes of humans and animals are not a true extravascular population, since parasites observed in lymphoid organs are located in the blood and lymph fluid rather than the lymphoid tissue. Although pathological changes such as lymphadenomegaly and chronic lymphoid depletion are commonly reported in HAT and AAT, these lesions are more likely due to the systemic immune response and immune exhaustion than direct damage by tissue-resident parasites [3,5].

### Cardiac and skeletal muscle

Cardiac symptoms are frequently recorded in HAT patients; up to 71% of HAT patients exhibit cardiac arrhythmias during chronic diseases, while around 20% show evidence of heart failure [42]. Although extravascular trypanosomes have not been observed in the cardiac tissue of human patients, myocarditis has been reported in autopsies of HAT patients [31,43,44]. In addition, cardiac-resident parasites have been well documented in both natural hosts and animal models of *T*. *brucei* infection [42,45].

In animal hosts, cardiac-resident parasites are commonly seen in all layers and structures of the heart, resulting in a lymphohistiocytic pancarditis and necrosis that results in cardiac damage and reduced cardiac function. Notably, in cattle, sheep, and dogs experimentally infected with Tbb, parasites seem to have a particular tropism for the atrial myocardium rather than the ventricular myocardium [7–9]. In Tbb-infected cattle, the largest number of parasites and most severe inflammation were present in the atrial myocardium, although extravascular parasites were observed throughout the heart [9]. Similarly, extravascular trypanosomes were present throughout the hearts of Tbb-infected sheep, with more severe associated inflammation in the atria than in ventricles [7]. In Tbb-infected dogs, parasites were detected in the atrial tissues at earlier time points than in the ventricles, although parasites and severe associated inflammation were found throughout the entire heart by end-stage disease [8]. In contrast to other large mammal hosts, cardiac-resident parasites in nonhuman primates have not been shown to exhibit a tropism for the cardiac atria, although trypanosomes have been observed in the cardiac valves of vervet monkeys experimentally infected with Tbr and Tbb [29].

In rodent models of African trypanosomiasis, cardiac-resident parasites and associated inflammation appear rapidly after infection and may preferentially target the cardiac atria, as in large animal models. The mechanisms of cardiac damage have not been well elucidated but likely represent a combination of direct parasite action and secondary inflammation. In Tbb-infected mice, extravascular parasites initially localized to the atrial endocardium and epicardium but became widespread in all layers and structures of the heart over time [46]. Interestingly, infection of immunosuppressed mice with Tbb resulted in the presence of cardiac-resident trypanosomes without associated inflammation or cardiomyocyte necrosis, suggesting that inflammation is at least partially responsible for the cardiac symptoms seen clinically [47]. Recently, the rat has been developed as a model of cardiac arrhythmias in HAT [48]. In rats experimentally infected with Tbb, cardiac-resident parasites were concentrated at the atrioventricular junction, in contrast to some other animal models in which parasites preferentially target the atria [48]. Interaction of Tbb parasites with ex vivo rat cardiomyocytes led to arrhythmias, suggesting that *T*. *brucei* has direct arrhythmogenic effects, which could cause symptoms of heart disease [48]. This is in contrast to findings in mice, in which the primary driver of cardiac damage and dysfunction seemed to be the host inflammatory response.

Overall, findings in humans, natural hosts, and rodent models demonstrate that the heart is an important and clinically relevant focus of extravascular trypanosomes. Following infection of animals, trypanosomes rapidly enter the heart, where they may have a particular affinity for the cardiac atria, although this affinity has not been demonstrated in all mammalian hosts. The reasons for this possible atrial affinity are uncertain. The cardiac atria contain higher levels of collagen than the ventricles, which may play a role in targeting trypanosomes to the atria, since trypanosomes in multiple tissues have an apparent tropism for collagen bundles [49]. Cardiac-resident parasites are associated with inflammation, which affects all layers of the heart and may contribute to cardiac symptoms via direct parasitic action or the host inflammatory response.

In addition to cardiac muscle, trypanosomes may reside in skeletal muscle. Unfortunately, despite evidence of muscular involvement in multiple animals, the skeletal muscle has remained largely unexplored as a potential tissue reservoir. Muscle pain, though a relatively nonspecific symptom, is reported in up to 20% of HAT patients and has been observed in dogs experimentally infected with Tbb [8,22,50]. Myositis has been reported in autopsies of HAT patients and has been observed to be especially prominent in the diaphragm [51]. In experimental infection of cattle, sheep, and dogs with Tbb, extravascular parasites and associated inflammation have been observed in skeletal muscle [7–9]. In sheep, Tbb parasites appeared to primarily be associated with the endomysium, perimysium, and epimysium—the collagen sheaths surrounding muscle bodies [7]. The anatomic distribution and the biological relevance of trypanosomes in the skeletal muscle in other animals remain to be fully elucidated.

### Adipose tissue

There is abundant evidence that the adipose tissue is important to the pathogenesis of trypanosomiases. Severe weight loss is a common manifestation of chronic African trypanosomiasis in both humans and animals and is recorded in up to 40% of human cases [5,22,52]. Weight loss is a nonspecific symptom, and the underlying mechanisms of weight loss in both humans and animals remain poorly understood. Weight loss could occur secondary to neurological disease, but recent evidence suggests that adipose-resident parasites may also play a role in weight loss by directly utilizing the host’s fat stores and inducing adipose tissue inflammation [20].

While data on adipose tissue–resident parasites in natural hosts is limited, experimental infections of cattle, sheep, and dogs with Tbb have shown that extravascular parasites commonly occupy the subcutaneous fat, where they are associated with cellulitis [7–9]. Unfortunately, investigation of the adipose tissues in natural hosts has been limited to the subcutaneous fat, and the distribution of adipose-resident parasites in other adipose depots, such as the gonadal, mesenteric, or perirenal fat, has not been determined in large-animal models.

One recent study has suggested that the adipose depots are a major tissue reservoir of *T*. *brucei* in mice [20]. In this study, Tbb parasites were found in multiple white and brown fat depots of experimentally infected mice at the earliest time points analyzed (6 days postinfection) [20]. Electron microscopy confirmed that parasites in fat were extravascular, primarily located between adjacent adipocytes or between adipocytes and blood vessels [20]. These parasites exhibited unique patterns of gene expression and demonstrated enhanced ability to use fatty acids as a carbon source, suggesting that *T*. *brucei* is able to modify its metabolism to take advantage of the adipose tissue niche [20]. Along with morphological changes that have been observed in skin- and brain-resident parasites, this could suggest more specific adaptation to extravascular niches than had previously been appreciated [12,53].

### Lungs

Pneumonia has been reported in humans and animals infected by *T*. *brucei*, and respiratory symptoms are recorded in up to 20% of HAT patients [5,7,54]. Respiratory symptoms are commonly attributed to secondary bacterial infection, however, and the lung has not been a major subject of *T*. *brucei* research. While experimental evidence suggests that it is possible for *T*. *brucei* to invade pulmonary tissues, evidence for such invasion is limited to rodent models.

Extravascular lung parasites were recorded at least as early as 1912, when Simeon Wolbach observed parasites in the peribronchial and perivascular lung tissue of rats and guinea pigs experimentally infected with Tbg, with associated interstitial pneumonia [6]. In one study, extravascular parasites were detected in the lung of mice experimentally infected with Tbb as early as 6 days postinfection via PCR and were more abundant in the lung than in the heart and brain at both 6 and 28 days postinfection, suggesting that the lung potentially is a site of early parasite tissue invasion and proliferation [20]. Bioluminescence imaging has detected parasites in the lungs of mice experimentally infected with Tbg during the early cryptic phase of infection, when vascular parasites are undetectable [55]. In contrast to these findings, a recent study using intravital microscopy has shown that the most parasites in the lung of mice experimentally infected with Tbb remain intravascular during early infection [56]. Together, these findings suggest that parasites in rodents have a tropism for the lung during early infection, although they may remain intravascular. Lung-resident parasites, which do invade the lung parenchyma, may also persist during later infection.

Though lung invasion is generally well supported in rodent models, lung-resident parasites have not been observed in large mammal hosts of *T*. *brucei*. It is possible that a unique anatomic feature of the rodent lung predisposes it to parasitic invasion: Rodent pulmonary veins contain a layer of cardiac muscle cells not found in other mammals [57]. Given *T*. *brucei’s* proclivity for the heart, these cardiomyocytes might encourage pulmonary invasion. To date, the pulmonary space remains largely uncharacterized as a possible anatomic reservoir for *T*. *brucei*, and the clinical relevance of the lung in natural infection remains to be determined.

### Endocrine glands and reproductive organs

Symptoms related to the endocrine and reproductive systems are frequently reported in HAT. Symptoms consistent with endocrine disturbances are reported in 16% to 50% of HAT patients [27,58]. In both men and women, HAT can be associated with decreased levels of sex hormones. In one survey, HAT was associated with menstrual cycle disturbances in 60% of women and impotence in 70% of men [3,59]. Similarly, reproductive failure and abortion are common sequelae of AAT [60,61]. Multiple causative factors have been implicated in endocrine and reproductive dysfunction during HAT and AAT, including direct inflammatory damage to endocrine organs, increased levels of circulating cytokines, and disruption of the hypothalamic–pituitary axis secondary to neurological disease [58,62,63]. Whether invasion of endocrine and reproductive organs by *T*. *brucei* also contributes to these symptoms is not clear. However, in experimental animal models, there is some evidence that parasites invade the pituitary gland, the testes, and female reproductive organs.

The majority of data on endocrine involvement in *T*. *brucei* infection come from experimental infection of sheep and dogs with Tbb. In both animal models, the most severely affected endocrine organ has been the pituitary gland. In both sheep and dogs, large numbers of parasites have been observed in the neurohypophysis, the region of the pituitary gland composed of nervous tissue [8,63]. Given *T*. *brucei*’s neurological tropism, the presence of parasites in the neurohypophysis is unsurprising. Parasites have also been observed in the adenohypophysis, the secretory epithelial region of the pituitary gland in sheep and dogs [8,63]. In both regions, extravascular parasites were accompanied by severe lymphoplasmacytic inflammation and destruction of normal pituitary tissue [8,63]. It has been suggested that pituitary gland damage could contribute to symptoms of HAT and AAT, but such damage has not been definitively demonstrated in human infection [58,63].

Involvement of the reproductive organs appears to be most common in the testes and associated male reproductive tissues. In male sheep, cattle, rabbits, and dogs experimentally infected with Tbb, extravascular trypanosomes have been observed in the tunica albuginea and pampiniform plexus, connective tissue structures associated with the testes [7–9]. In one mouse study, parasites were observed in the testicular connective tissue at the earliest experimental time point (6 days postinfection with Tbb) [17]. Notably, parasites were found solely in the connective tissue and had not crossed the blood–testis barrier, which separates the seminiferous tubules from the rest of the testes. Foci of severe inflammation and tissue damage were associated with degenerating parasites [17]. While it has been suggested that the blood–testis barrier could protect testis-resident parasites from host immunity and chemotherapeutic agents, parasites in both natural hosts and mice primarily localize outside of the blood–testis barrier, and there is evidence for a strong immune response against testes-resident *T*. *brucei* [17,64].

Although abortion and failure to breed are common clinical signs of AAT, parasitic invasion of the female reproductive organs has not been definitively established. Lesions of the ovaries and uterine wall were reported in dogs experimentally infected with Tbb, although the nature of the lesions and the presence of extravascular parasites were not reported [8]. A recent study in mice experimentally infected with Tbg demonstrated trypanosome DNA and bioluminescent parasites in the ovaries and uterus, even in the absence of detectable parasitemia in the blood, suggesting that the female reproductive organs may be an anatomic reservoir of parasites during latent infection [65]. The possibility that the female reproductive organs may be an anatomic reservoir for trypanosomes is further supported by the presence of detectable parasite DNA in the ovaries of goats experimentally infected with *Trypanosoma vivax*, an African trypanosome related to *T*. *brucei* [66].

Intriguingly, recent research using Tbg in mice has suggested that *T*. *brucei* in the reproductive organs may be horizontally and vertically transmissible. Male mice experimentally infected with Tbg were able to transmit infection to healthy female mice, in which trypanosomes were subsequently detected in the ovary, uterus, and CNS [65]. Experimentally infected female mice were also able to transmit infection to both male and female offspring [65]. However, aside from a single case of possible sexual transmission of Tbg in a human, there is little evidence of horizontal transmission in natural hosts [67]. Vertical transmission occurs in humans but is thought to be rare. Only 13 instances of Tbg and a single instance of Tbr vertical transmission have been recorded [68]. The involvement of gonadal tissue–resident parasites in such cases of direct transmission is not known. Overall, there is evidence that *T*. *brucei* parasites can invade endocrine and reproductive organs, but the clinical and biological relevance of this invasion is still unclear.

### The eye

Ocular symptoms have been reported in up to 30% of HAT cases [16]. Unfortunately, the clinical picture may be complicated by concurrent eye disease, and a detailed ophthalmic exam is often not performed on HAT patients, making it challenging to determine whether ocular symptoms are due to trypanosomiasis, adverse effects of antitrypanosomal drugs, or unrelated conditions [16].

Ocular involvement in African trypanosomiasis has mostly been investigated in large animals. In cats, sheep, and dogs experimentally infected with Tbb, parasites have been observed in the ciliary body, in association with mononuclear inflammation [8,10,11]. These findings indicate that trypanosomes may enter the eye via the ciliary body, where blood is filtered to produce aqueous humor. Interestingly, the ciliary body shares a common embryological origin with the choroid plexus, where trypanosomes are thought to cross the blood–cerebrospinal fluid (CSF) barrier, so this route of entry is theoretically plausible [3,69]. Aside from the ciliary body, Tbb parasites in experimentally infected sheep and dogs have been observed throughout the eye, in association with severe lymphoplasmacytic panophthalmitis, inflammation of the entire eye [8,11].

Findings in donkeys and goats suggest that parasites can persist in the aqueous humor long term and that some eye-resident trypanosomes may be able to escape drug treatment. In donkeys experimentally infected with Tbb, trypanosomes were observed in the aqueous humor 2 months after infection, suggesting that the eye may represent an anatomic reservoir for trypanosomes [70]. In goats infected with *T*. *vivax*, the presence of extravascular trypanosomes in the aqueous humor was associated with a relapse of parasitemia after chemotherapy, suggesting that the eye could serve as a refuge from trypanocides, although this has not yet been demonstrated during infection with *T*. *brucei* [71].

To date, there is no rodent model of ocular African trypanosomiasis, despite the possible clinical importance of eye-resident parasites in human disease. Development of a rodent model for ocular African trypanosomiasis would allow further investigation of the eye as a site of disease and parasite persistence.

### Common themes and species differences

Unsurprisingly, there are some differences in extravascular parasites between different mammalian hosts. An understanding of these differences can inform the use of animal models to interrogate specific questions. Of the natural hosts, canines appear to exhibit the largest numbers of extravascular parasites, with the most severe and rapidly progressive tissue pathology [8]. Rabbits exhibit ulcerative dermatitis and granulomatous inflammation in response to extravascular parasites, which is distinct from the lymphoplasmacytic and histiocytic inflammation seen in most other animals [40]. In rodents, parasites seem to have an affinity for the lung, which has not been demonstrated in other hosts.

Perhaps the greatest species difference lies between humans and all other natural and experimental hosts: Although extravascular parasites have been described in a wide variety of naturally and experimentally infected animals, they are rarely observed in human autopsies and biopsies. This discrepancy may be partially explained by the relatively low number of human tissue samples available for study and the challenge of preserving human tissues to avoid parasite degradation [43]. In addition, treatment with antitrypanosomal drugs may disrupt the natural course of infection and eliminate extravascular parasites. Finally, in animal models, extravascular parasites have most often been studied during the acute phase of infection, when parasitemia is high. In contrast, most human cases are caused by Tbg, with consequent low parasitemia. If there is a correlation between the abundance of parasites in the blood and extravascular tissues, low parasitemia could at least partially explain the rarity of extravascular parasites in human biopsy and autopsy samples.

Of course, apparent differences in pathology between animal hosts must be interpreted with caution: Experimental methods often vary drastically between animal studies, including the *T*. *brucei* strain used, infection route, experimental endpoints, tissues collected, and analyses performed. Despite these many variables, common themes emerge across the spectrum of mammalian hosts. Distributions of tissue-resident parasites and pathological findings are relatively consistent. In all hosts and organs, *T*. *brucei* appears to have an affinity for collagenous connective tissue, a tropism that has been noted in several animal models [7,12,29,30]. Regardless of the organ they are invading, extravascular parasites most often localize to regions rich in collagen, where they are noted to disrupt and occupy collagen bundles: the dermis, skeletal muscle sheaths, cardiac atria, serous membranes, and adipose depots. The underlying reasons for this possible collagen affinity are not understood.

In all hosts and organs, extravascular parasites persist in tissues despite a strong inflammatory response, resulting in organ damage and loss of function. Generally, the inflammatory response is characterized by lymphoplasmacytic and histiocytic infiltrates in tissues. The severity of inflammation is often correlated with the abundance of extravascular parasites, although inflammation occasionally occurs in the absence of detectable extravascular parasites. Initially, tissues exhibit degeneration and necrosis, with replacement of tissue by nonfunctional scar tissue over time. Both inflammation and chronic fibrosis in tissue spaces may contribute to clinical symptoms. Overall, findings suggest that tissue-resident trypanosomes induce severe inflammation, which results in clinically significant tissue damage but is nevertheless unable to effectively clear parasites. The specific nature of the extravascular immune response to *T*. *brucei* remains to be characterized.

### Advantages of tissue tropism

What role does tissue tropism play in *T*. *brucei* biology and pathogenesis? At first glance, establishment of tissue-resident parasites in any space besides the dermis appears counterintuitive to the needs of the parasite. *T*. *brucei* relies on transmission to the tsetse fly to complete its life cycle. Extravascular parasites represent a biological dead end unless they reenter the vasculature or target the dermis where they can be transmitted. While it is possible that extravascular parasites are incidental to *T*. *brucei* biology, there are several possible reasons why tissue tropism may bestow an active advantage to *T*. *brucei* parasites.

First, tissue tropism may protect parasites from chemotherapeutics. Such an advantage is likely incidental, rather than a specific reason why parasites evolved tissue tropism, given that tissue tropism exists in *T*. *brucei* strains, which have not been exposed to chemotherapeutics. It is unclear how well antitrypanosomal agents penetrate tissues besides the brain. The eyes, brain, and adipose tissue, however, are all occupied by extravascular parasites and are well known for their impermeability to multiple drugs [72–74]. Parasites in the aqueous humor of the eye have been implicated in relapse of *T*. *vivax* in goats following chemotherapy, providing evidence that extravascular parasites may be protected from some drugs and capable of reseeding the vasculature [71]. However, evidence of similar recurrence has not been demonstrated in *T*. *brucei* infection.

Second, tissue tropism may allow the utilization of unique energy resources. There is recent evidence that Tbb parasites within adipose tissue adapt to use fatty acids as a carbon source and that Tbb parasites within artificial human skin modify their transcriptome to adapt to the skin microenvironment [20,33]. These findings suggest that *T*. *brucei* adapts to take advantage of specific tissues. Using tissue-specific energy sources could allow extravascular parasites to tap into energy resources that are not available to vascular parasites.

Third, extravascular parasites may facilitate immune evasion and provide a reservoir to reestablish the population of vascular parasites. *T*. *brucei* parasites undergo antigenic variation of their variant surface glycoprotein (VSG) coat, and individual antigenic types are typically cleared before isotype switching occurs [75]. Thus, the antibody response to *T*. *brucei* is primarily dependent on immunoglobulin M (IgM), the bulkiest antibody isotype. IgM may be slower to penetrate tissues and less capable of passive diffusion into tissues than smaller antibody isotypes, making it potentially less capable of clearing extravascular parasites [76–78]. Indeed, one study in rabbits found fewer anti-Tbb antibodies in connective tissue fluid compared to serum, suggesting that the antibody response could be less effective at clearing parasites in connective tissue than in blood [79]. Extravascular parasites may also express distinct VSGs from vascular populations, diluting the systemic antibody response to any one VSG and accelerating immune exhaustion. It is also possible that parasites occupy immune-privileged sites such as the eye to evade host immunity. However, the presence of parasites in nonimmune-privileged tissues suggests that any affinity for immune-privileged sites is unlikely to be a major mechanism of immune evasion. More generally, it is well established that tissue spaces exhibit distinct immune responses, and this could lead to differential survival among parasite populations throughout the body [80]. Overall, tissue-resident parasites may exhibit enhanced survival due to the unique immune milieu of each tissue space.

Of course, it is also possible that individual extravascular parasite populations may serve unique purposes in the life cycle of *T*. *brucei*. For example, some tissue-resident parasite populations may be important for transmission. There is evidence that skin-resident parasites may be crucial to long-term transmissibility, while gonadal tissue–resident parasites may also play an underappreciated role in vertical and horizontal transmission [18,33,35,65]. Other tissue-resident populations may contribute to immune evasion, constituting reservoirs that are able to reseed the vasculature in the face of the host adaptive immune response. It has been suggested that tissue-resident parasites in the adipose bodies and eyes may be less exposed to host immunity compared to bloodstream parasites and that these organs may serve as a refuge from the host response during chronic infection [20,71]. To date, though, the roles that different tissue-resident populations play in the *T*. *brucei* life cycle remain poorly characterized, and it is unknown how these populations relate to one another.

### Future questions

More than a century of research involving multiple animal models has provided abundant evidence that *T*. *brucei* parasites invade and reside in extravascular spaces. These tissue-resident parasites are capable of surviving and proliferating in a wide variety of niches, with a possible affinity for collagenous connective tissue. Although their role in clinical disease has not been thoroughly researched, tissue-resident parasites and associated tissue damage have been associated with several extra-CNS manifestations of African trypanosomiasis, including heart disease, weight loss, endocrine dysfunction, reproductive failure, and vision loss. Parasite entry and exit from tissues may also be partially responsible for the vasculitis and hemorrhage, which are occasionally reported in both HAT and AAT [31,81]. However, although recent interest in extravascular parasites has led to increased research, our understanding of extravascular parasites remains limited. As they become an increased focus of research in the future, several important questions remain to be answered.

What role do extravascular parasites play in the biology and life cycle of *T*. *brucei*? Extravascular spaces in the dermal matrix represent the initial site of parasite invasion and proliferation following infection and may play an important role in transmission during long-term infection [18,23]. In addition, there is evidence that parasites exit the bloodstream to enter extravascular tissues as rapidly as 6 days postinfection in mice and that CNS invasion may occur earlier than previously appreciated [82]. The timeline of parasite entry into tissues and whether this process is continuous or temporally restricted are unknown. It is also uncertain whether extravascular parasites are capable of reentering the vasculature. Reentrance of extravascular parasites into the bloodstream could allow *T*. *brucei* to reestablish parasitemia in the face of a strong antibody response and provide a better chance of passage to a tsetse fly vector. Reseeding of the vasculature by extravascular parasites could also explain some cases of treatment failure. However, whether reentry into the vasculature occurs, as well as the mechanisms of such reentry, remain to be determined.

How does *T*. *brucei* exit the vasculature and enter tissues? There are several host factors that are believed to contribute to *T*. *brucei* entry into the CNS. Although mechanisms of extra-CNS entry have not been investigated, it is probable that comparable factors are involved. Presence of LAMA4 and absence of LAMA5 may direct parasites to extravasate at specific locations [83]. The adhesion molecules ICAM-1, VCAM-1, CD36, and E-selectin have also been implicated in CNS and extra-CNS invasion [56,84,85]. On the parasite side, there has been little research on *T*. *brucei-*specific factors involved in tissue invasion. The *T*. *brucei* protease, Brucipain, may facilitate parasite extravasation by increasing endothelial permeability [86]. In vivo imaging of *Trypanosoma carassii*, a distantly related trypanosome, in zebra danio has suggested that the factors responsible for trypanosomal endothelial adhesion may be spatially restricted to the parasite’s posterior end [87]. An improved understanding of *T*. *brucei’s* surface protein distribution could help identify hypothetical adhesion proteins.

What factors cause *T*. *brucei* to target specific organs and tissues? In *Trypanosoma cruzi*, the FLY peptide of gp85/*trans*-sialidase glycoproteins may play a role in cardiac tropism by binding to intermediate filaments on cardiac endothelium [88]. Similarly, PfEMP1 in *Plasmodium falciparum* mediates the adhesion of infected erythrocytes to specific endothelial compartments [89]. If there are analogous peptides or proteins in *T*. *brucei*, they could similarly confer tissue tropism by recognizing organ-specific endothelial proteins. In addition, it is possible that *T*. *brucei* parasites utilize chemotactic sensing of organ-specific molecules or metabolic products to recognize and target specific organs. Quorum sensing and chemotaxis have been demonstrated in *T*. *brucei*, so it is possible that *T*. *brucei* could target extravascular spaces by recognizing and moving toward organ-specific molecules [90,91]. However, the role of chemotaxis in tissue invasion has not been investigated. On the host side of the equation, differing expression of adhesion molecules and laminins on vasculature of tissues could influence tissue tropism, since these proteins have been implicated in *T*. *brucei* extravasation.

What is the proliferative capacity of extravascular parasites, and do they differentiate into transmissible stumpy forms outside of the vasculature? This question is central to understanding whether extravascular parasites play a role in transmission, potentially contributing to an asymptomatic reservoir of HAT. Experiments using an artificial human skin model suggest that Tbb parasites in the dermis rapidly differentiate to quiescent forms that are able to survive long term without inducing inflammation, creating a reservoir that could continuously reseed the vascular compartment and/or contribute to transmission in asymptomatic patients [33]. Supporting this idea, another recent study identified extravascular parasites in the dermis of 6 previously undiagnosed individuals, including transmissible stumpy forms in the dermis of 1 patient, suggesting that a potentially transmissible skin-resident population remains even when parasites are absent from the blood [18]. While there is evidence that adipose-resident parasites differentiate into stumpy forms, the proliferative capacity and differentiation of other populations of tissue-resident parasites remain to be characterized [20].

How does *T*. *brucei* cause tissue damage? Tissue-resident parasites are associated with damage to a number of organs. However, the relative contribution of direct parasitic action versus host inflammation has not been determined. A switch from a pro-inflammatory to anti-inflammatory macrophage phenotype in chronic disease is associated with an improved outcome, and immunodeficient mice exhibit less severe cardiac pathology, suggesting that pathological inflammation is responsible for at least some tissue damage [46,92]. However, recent findings that trypanosomes can directly disrupt connective tissue, generate cardiac arrhythmias, and use adipose stores as an energy source suggest that the picture is more complex and that parasites are responsible for at least some direct tissue damage [20,48,87].

Do tissue-resident parasites differ between mammalian hosts and *T*. *brucei* subspecies? Although pathological findings are relatively conserved, there are some possible variations between animal hosts. For example, canines exhibit unusually severe and widespread tissue pathologies, while rabbits exhibit unique inflammatory responses [8,40]. It is also possible that tissue tropism varies between *T*. *brucei* subspecies, since Tbg and Tbr cause distinct clinical syndromes. However, any differences in pathology and tissue tropism between *T*. *brucei* subspecies have not been well characterized. Appreciating the potential differences in tissue tropism between mammalian hosts and parasite strains could help inform development of better research models for HAT and AAT.

*T*. *brucei* is both a hemolymphatic and a tissue parasite. Recent research has led to an increased appreciation for the roles that non-CNS tissue-resident parasites play in the biology of *T*. *brucei* and the pathogenesis of HAT and AAT. Extravascular parasites may target specific organs and tissues, evade the immune response in unique ways, contribute to treatment failure, change their metabolism to exploit tissue-specific host resources, and directly or indirectly cause tissue damage. Despite recent progress in our understanding of tissue-resident parasites, many questions remain to be answered. Renewed interest in this subject will hopefully lead to answers to many of these questions.
